# *Curcurbita pepo* subspecies delineates striped cucumber beetle (*Acalymma vittatum*) preference

**DOI:** 10.1038/hortres.2016.28

**Published:** 2016-06-15

**Authors:** L Brzozowski, B M Leckie, J Gardner, M P Hoffmann, M Mazourek

**Affiliations:** 1 Section of Plant Breeding and Genetics, School of Integrative Plant Science, Cornell University, Ithaca, NY, USA; 2 School of Agriculture, Tennessee Tech University, Cookeville, TN, USA; 3 Department of Entomology, Cornell University, Ithaca, NY, USA

## Abstract

The striped cucumber beetle (*Acalymma vittatum* (F.)) is a destructive pest of cucurbit crops, and management could be improved by host plant resistance, especially in organic farming systems. However, despite the variation in striped cucumber beetle preference observed within the economically important species, *Cucurbita pepo* L., plant breeders and entomologists lacked a simple framework to classify and exploit these differences. This study used recent phylogenetic evidence and bioassays to organize striped cucumber beetle preference within *C. pepo*. Our results indicate preference contrasts between the two agriculturally relevant subspecies: *C. pepo* subsp. *texana* and *C. pepo* subsp. *pepo*. Plants of *C. pepo* subsp. *pepo* were more strongly preferred than *C. pepo* subsp. *texana* plants. This structure of beetle preference in *C. pepo* will allow plant breeders and entomologists to better focus research efforts on host plant non-preference to control striped cucumber beetles.

## Introduction

The damage inflicted upon plants of the Cucurbitaceae family by the striped cucumber beetle, *Acalymma vittatum* (F.) (Coleoptera: Chrysomelidae), is a well-studied and economically important phenomenon.^1–8^ Striped cucumber beetles cause significant damage to cucurbit crops (squash, pumpkin, watermelon, cucumber and melon) via herbivory of foliage, flowers, fruit and roots,^4^ and by vectoring pathogens of major diseases like bacterial wilt (*Erwinia tracheiphila*)^9^ and *Squash mosaic virus.* ^10^ All cucurbit crops are affected by these beetles.

The relative preference of striped cucumber beetles—and thus degree of economic damage—varies within the Cucurbitaceae family.^5,6,11^ Because striped cucumber beetles can devastate a newly planted crop,^4^ some control strategies have been developed. For instance, growers using conventional methods have access to effective chemical controls, in particular, systemic neonicotinoid insecticides.^12^ However, use of these pesticides has come under scrutiny because of their potential impact on pollinator health.^13–15^ Cultural controls, such as row covers and trap cropping are also advised to growers, but they do not offer complete control and add expense, like labor, materials or loss of production space to a trap crop.^16^ One avenue for optimizing control of these beetles would be to elucidate mechanisms behind what drives striped cucumber beetle preference, and to exploit those variations in preference to develop effective plant-based control.

The underlying biology influencing herbivore preference within the Cucurbitaceae family has been extensively studied for a range of cucurbit species and a group of specialist diabroticite beetles (Chrysomelidae: Galerucinae: Luperini) to which striped cucumber beetles belong. It has been broadly established that cucurbitacins, bitter tetracyclic triterpenoids, are toxic to most generalist herbivores, but are feeding stimulants to this tribe of beetles.^11,17,18^ However, *A. vittatum* has been shown to be the least responsive to cucurbitacins of this group of beetles.^18,19^Although *A. vittatum* adults do choose to feed, and larvae perform better, on cucumber (*Cucumis sativus*) plants with functional production of cucurbitacin C,^1,20^ they do not require cucurbitacin C for key physiological processes, like production of their aggregation pheromone,^21,22^ and the feeding response elicited by cucurbitacin C is highly dependent on their life history.^23^ Other leaf chemistry or nutrition could also influence preference.^20,24^ In addition, interspecific differences in plant volatiles also have a role in attractiveness.^3,25–27^

There are systems where hypotheses about the role of these biochemical factors driving preference can be tested. For instance, isogenic lines of cucurbitacin C producing and non-producing lines of cucumber are available.^28^ However, one species where such resources have not existed is the economically important *Cucurbita pepo* species.

*C. pepo* houses two agriculturally important and genetically diverse subspecies *C. pepo* subsp. *pepo*, and *C. pepo* subsp. *texana,*^29,30^ that likely arose from distinct domestication events.^31–33^ These subspecies include a variety of different market classes of squash: *C. pepo* subsp. *pepo* includes cocozelle, pumpkin, vegetable marrow and zucchini, whereas *C. pepo* subsp. *texana* includes acorn, crookneck, scallop and straightneck squash.^34^ These market classes are comprised of a phenotypically diverse array of crops,^35^ as some are eaten as immature fruit (for example, zucchini), while others are eaten as fully mature fruit (for example, acorn squash).

Importantly, variation in striped cucumber beetle host preference has been observed within this species.^5,6,11,36^ However, there does not yet appear to be a singular metabolite predictor of striped cucumber beetle preference in *C. pepo*. Instead, studies have indicated that there may be a myriad of factors that contribute to striped cucumber beetle preference within *C. pepo*, including beetle life history,^23^ aggregation pheromones,^21,22^ nutrition^20,24^ and variation in cucurbitacins levels.^11^

Because of the abundance of complex relationships that drive preference, it would be valuable to develop a simple framework for organizing striped cucumber beetle preference within *C. pepo*. Having an established framework could unify studies on factors affecting beetle preference and allow for these to be more easily incorporated in plant breeding decisions.

Accordingly, the objective of this study was to structure *A. vittatum* preference in *C. pepo*. A wide variety of cultivated *C. pepo* varieties, including those of the different subspecies and market classes, were tested in greenhouse and field choice assays to evaluate host preference of striped cucumber beetles. In addition, farm-scale field and greenhouse no-choice studies were performed to further elucidate host preference traits in *C. pepo* and inform deployment strategies of non-preference traits.

## Materials and methods

### Plant material

A panel of 29 cultivars from six *C. pepo* market classes cultivated for harvest of immature fruits (Table 1), hereafter ‘early-harvest panel’, and a survey of 27 *C. pepo* cultivars from five market classes, including those harvested as immature and mature fruits, (Table 2), hereafter ‘mixed-harvest panel’, were used in cultivar choice trials. In no-choice bioassays, one inbred cultivar from each subspecies that represented the phenotypic extremes of beetle preference, as determined by the panel surveys, was grown: Golden Zucchini (*C. pepo* subsp. *pepo*) as the highly preferred cultivar, and Success PM straightneck summer squash (*C. pepo* subsp. *texana*) as the highly non-preferred cultivar. Plants for all bioassays were started from seed in Fort Light potting soil (Vermont Compost Company, Montpelier, VT) in the Cornell University Guterman Bioclimatic and Greenhouse Complex (Ithaca, NY), and bioassays were conducted when plants had two or more leaves, but had not yet flowered.

### Insects

All adult striped cucumber beetles (*A. vittatum*) were obtained from the Organic Research Farm (Freeville, NY, USA) managed by the Cornell University Agricultural Experiment Station. The field trials took advantage of naturally occurring beetle populations in fields, which had been planted to representatives of every genus, species and subspecies of most commonly cultivated melons, watermelons, squash and cucumbers. Two adult generations of striped cucumber beetles occur in this region;^4^ all trials except the no-choice greenhouse trial used first generation adult beetles (beetles that emerged from overwintering in Spring 2014 or Spring 2015).

### Field cultivar bioassays

Field bioassays of both cultivar panels were conducted at Freeville Organic Research Farm over two consecutive years (2014–2015). Seeds for the 2014 early-harvest panel field trial were sown in 50 cell trays in a greenhouse on 27 May 2014, and transplanted into the field on 24 June, 2014. Seeds for the 2015 mixed-harvest panel field trial were sown in 72 cell trays in a greenhouse on 26 June 2015, and transplanted into the field on 7 July 2015. In both the years, plants were hardened off in a cold frame and then transplanted into soil covered with black plastic mulch with 2.7-m spacing between the rows. Trials were arranged in a randomized complete block design with each of five replicates containing three plants planted 0.3-m apart, and cultivars separated by 0.9 m, within rows. The soil was amended with compost to achieve recommended fertility levels for these crops. Trained scorers evaluated beetle damage by visual assessment once damage was evident by using a non-linear 1–5 scale for damage (1=0–10%, 2=11–30%, 3=31–60%. 4=61–90%. 5=91–100%). Backtransformation of this categorical data for analysis was then performed by using the mean percent defoliation of the range representing the categorical classification (for example, 5% and 20.5% were used for scores 1 and 2, respectively).

### Greenhouse cultivar bioassays

To complement the field bioassays, both cultivar panels were grown and evaluated in controlled conditions using enclosure cages at the Guterman Greenhouse. Seeds were sown into detachable nursery cell packs, and seeding occurred on 12 July 2014 for the mixed-harvest panel greenhouse trial and on 26 June 2015 for the early-harvest panel greenhouse trial. Three blocks were planted with four plants of each cultivar in a randomized complete block design, and placed within a 1×0.2×2 m spun-bound polyester cage (Agribon, San Luis Potosí, México) in which they were collectively exposed to beetles on 22 July 2014, and on 7 July 2015. Field collected beetles were added to the cages until sufficient damage was achieved, as in Barber *et al*.^37^ Both trials were terminated once damage was visibly evident and deemed significant, about 72 h after initial exposure to beetles. Individual leaves and cotyledons were destructively removed from every plant to be digitally imaged, and percent leaf defoliation was then calculated by measuring total leaf area and estimating missing leaf area in ImageJ.^38^

### Field no-choice bioassay

In addition to understanding the effect of subspecies on beetle preference given choice among a range of cultivars, the differences in herbivory elicited by subspecies when *A. vittatum* had no choice of food source were also explored. Previous greenhouse surveys with low numbers of plants (four to six plants) indicated that feeding behavior differed between ‘Golden Zucchini’ and ‘Success PM’ in a no-choice scenario (data not shown). To address the overarching goal of *A. vittatum* non-preferred cultivar development, a farm-scale no-choice bioassay was conducted. Accordingly, four large, 0.1 ha square (30.5 m×30.5 m) sites spaced at least 350 m apart were chosen at the Pullyen–Tailby Farm managed by the Cornell University Agricultural Experiment Station in Varna, NY. Each site was prepared as 10 equally spaced rows 30 m in length, covered with black plastic mulch, and managed organically. ‘Golden Zucchini’ and ‘Success PM’ seeds were sown into 50 cell flats in the Guterman Greenhouse between 1 and 2 June 2015, hardened off, and then transplanted into two field sites each at 0.6 m spacing (50 plants per row; 500 plants per site) on 17 June 2015. All plants were also fertilized on 22 June 2015 with Perdue AgriRecycle microSTART60 Plus with feather meal in 7-1-1- Prill form to achieve recommended crop fertility. Leaf damage was then scored visually as percent defoliation using a linear 0–100 scale by units of five (0=0%, 5=1–5% defoliation, 10=6–10% defoliation, and so on) for all plants in all plots by a single-trained observer beginning when *A. vittatum* was first observed in one plot, a ‘Golden Zucchini’ site, on 19 June 2015. Plots were scored approximately three times weekly until 17 July 2015, the point in the season when *A. vittatum* populations expectedly declined in our region.^4^ As there were no feeding *A. vittatum* populations observed in the two ‘Success PM’ sites for over 15 days after beetles were first observed in the first ‘Golden Zucchini’ site, they were supplemented with ~500 beetles collected from the Organic Research Farm (Freeville, NY, USA) on 6 July 2015. The addition of beetles to the ‘Success PM’ sites was done in attempt to assess whether damage would have occurred if striped cucumber beetles were present.

### Greenhouse no-choice bioassay

To control for field variability, and the ability of *A. vittatum* to leave the sites, large no-choice bioassays were conducted in the Guterman Greenhouse. ‘Golden Zucchini’ and ‘Success PM’ seeds were sown in 72-cell flats on 15 September 2015. Two flats of each cultivar were then placed in individual cages and exposed to field collected beetles per cage on 27 September 2015, and this was replicated twice (four total cages). Once damage was evident, on 9 October 2015, leaves were digitally imaged and scored for percent leaf damage.

### Statistical analysis

For all-choice bioassays, percent defoliation was analyzed by an analysis of variance performed in JMP Pro 11 (JMP, Version 11. SAS Institute Inc., Cary, NC, USA, 1989–2007) using a generalized linear model. Subspecies, market class and cultivar were treated as fixed effects with cultivar nested within market class, and market class nested within subspecies. The effects of blocking and replication were treated as random effects. Least squared means were separated using a *t*-test for testing between subspecies (*P*<0.05), and by Tukey’s HSD test for between market classes and cultivars (*P*<0.05.) To determine the distribution of beetle damage in the no-choice assays, data were grouped into two categorical bins—minimal leaf damage (field: <10%, greenhouse: 0%), and significant leaf damage (field: ⩾10%, greenhouse: >0%)—and Fisher’s exact test was used to test the null hypothesis that there was no difference in distribution of beetle damage between subspecies. These data were blocked by plot in the field trials, and cage in greenhouse trials. In addition, in the greenhouse no-choice assay, an additional categorical grouping of extreme (⩾80%) leaf damage was also examined by Fisher’s Exact test.

## Results

### Field cultivar bioassays

**Figure 1:**
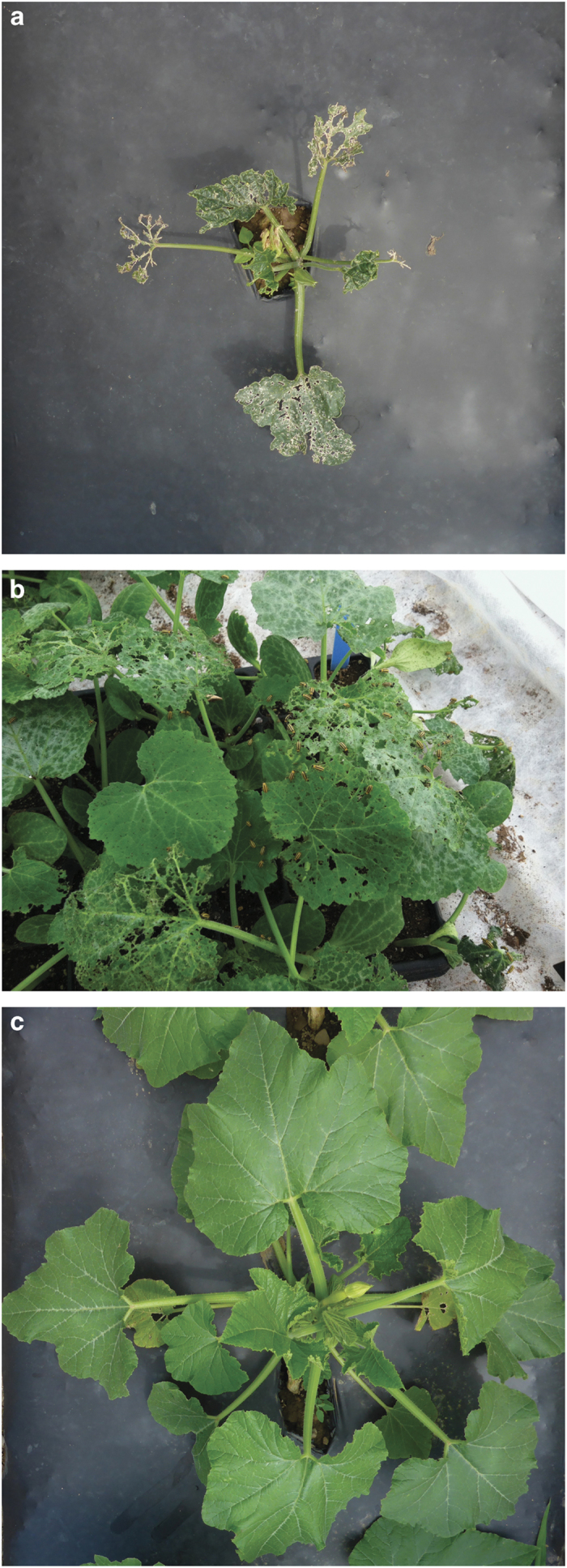
A comparison of adjacent young transplants in the field highlights the impact of striped cucumber beetle preference on plant health. *C. pepo* subsp. *pepo* cultivar ‘Golden Zucchini’ (**a**) is highly preferred and incurs substantial damage as the beetles aggregate and feed (**b**), whereas the non-preferred *C. pepo* subsp. *texana* cultivar ‘Success PM’ (**c**) is nearly free of herbivory damage.

Early-harvest and mixed-harvest panels were evaluated at the Organic Research Farm to test *A. vittatum* preference in a real-field setting, and it was found that transplanted seedlings of *C. pepo* subsp. *texana* suffered less herbivory damage by natural populations of striped cucumber beetles than plants of the other subspecies, *C. pepo* subsp. *pepo* (Figure 1). In the early-harvest panel, the damage observed within the *C. pepo* subsp. *texana* cultivars ranged from 5% defoliation of several straightneck and crookneck cultivars, including Success PM, to 10% defoliation of the straightneck cultivar Lioness (Table 1). In contrast, every *C. pepo* subsp. *pepo* cultivar had higher mean percent defoliation than those in *C. pepo* subsp. *texana*, with a range from 20% in zucchini cultivar Partenon to 46% in vegetable marrow cultivar Romulus. There were significant differences in leaf defoliation between all market classes in the different subspecies, as well as between subspecies as a whole (*P*<0.0001), with the degree of damage being more severe for *C. pepo* subsp. *pepo*. Likewise, in the mixed-harvest panel, expanded to include market classes of *C. pepo* harvested as mature fruit, there was again a significant difference in damage between subspecies (*P*<0.0001), which was also demonstrated within market class groupings (but not by individual cultivars; Table 2). The extremes of leaf defoliation in each subspecies within the mixed-harvest panel were 1 to 9% in *C. pepo* subsp. *texana* (‘Golden Bush Scallop’, and ‘Sweet REBA’, respectively), and 9 to 54% in in *C. pepo* subsp. *pepo* (‘Racer’, and ‘Golden Zucchini’, respectively).

### Greenhouse cultivar bioassays

To control for field variation, both panels were also exposed to field-collected striped cucumber beetles in a restricted greenhouse setting. The beetles caused significantly more beetle damage to the *C. pepo* subsp. *pepo* cultivars, echoing the results of the field trial. In the early-harvest panel, the difference in defoliation by subspecies was highly significant (*P*<0.0001; Table 1). The extremes of leaf defoliation in each subspecies were 1 to 5% in *C. pepo* subsp. *texana* (‘Lioness’, and ‘Slick Pik’, respectively), and 4 to 26% in *C. pepo* subsp. *pepo* (‘Harukan’, and ‘Zucchini Elite’, respectively). The mixed-harvest panel yielded similar results. Damage in *C. pepo* subsp. *texana* cultivars ranged from 0% defoliation of straightneck cultivar Early Prolific Straightneck to 10% defoliation of the acorn/delicata cultivar Honeyboat, whereas damage in *C. pepo* subsp. *pepo* ranged from 5% defoliation of pumpkin cultivar Magic Lantern to 22% defoliation of the zucchini cultivar Golden Zucchini (Table 2). Again, the difference in beetle damage between subspecies was significant (*P*<0.0001). In neither cultivar panel did market class or cultivar alone demonstrate as clear a significant divide between subspecies as did evaluating all the cultivars together.

### Field no-choice bioassay

**Figure 2:**
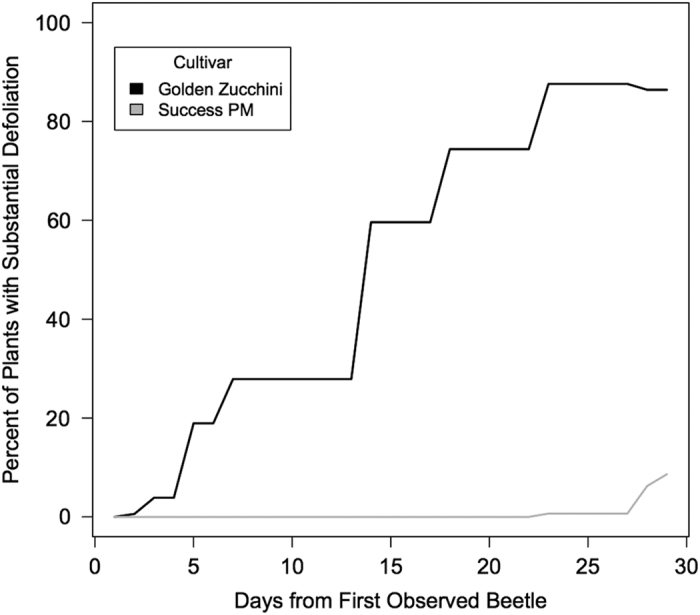
Leaf defoliation progress in field no-choice plots. Averaged number of plants with substantial (⩾10% leaf defoliation) in no-choice field plots. Difference between cultivars in leaf damage was significant (*P*<0.0001) at day 2, and continued to be at the final day of observation, day 29 (*P*<0.0001). *A. vittatum* were added to ‘Success PM’ plots on day 18, about 4 days before reflected by an increase in defoliated ‘Success PM’.

No-choice bioassays with cultivars representing the extremes of preference between subspecies were conducted to determine whether *C. pepo* subsp. *texana* (represented by straightneck cultivar ‘Success PM’) was not a suitable host, or whether *C. pepo* subsp. *pepo* (represented by zucchini cultivar ‘Golden Zucchini’) was just so strongly preferred that *C. pepo* subsp. *texana* was never fed upon in a mixed environment. These no-choice bioassays were also designed to understand the agricultural relevance of the non-preference phenotype, and its applicability in plant breeding: for instance, if non-preference could be introgressed into all *C. pepo*, particularly *C. pepo* subsp. *pepo* cultivars, then could non-preference be a stand-alone control measure at farm scale? Two farm-scale no-choice monocultures of ‘Success PM’ and two of ‘Golden Zucchini’ were observed. It was found that ‘Success PM’ plots had far more plants with <10% leaf defoliation than did the ‘Golden Zucchini’ plots (Figure 2; Fishers exact two-tailed test, *P*<0.0001). Individual plant leaf defoliation ranged from 5 to 90% in ‘Golden Zucchini’ plots, and from 0 to 15% in ‘Success PM’ plots on the final day of observation. In addition, the ‘Success PM’ plots also attracted no natural feeding populations of striped cucumber beetles (LB, personal observation). Accordingly, *A. vittatum* from the Organic Research Farm were added to ensure evaluation of beetle damage was possible. However, even with the addition of beetles to the ‘Success PM’ plots, very few beetles remained in those plots, and those that did inflicted very little damage.

### Greenhouse no-choice bioassay

As one limitation of the field no-choice experiment is that the beetles could not be contained, the field results were verified in a controlled greenhouse setting. There was no significant difference in the mean damage (total leaf defoliation) between cultivars in the greenhouse no-choice bioassay (Table 3), indicating that *C. pepo* subsp. *texana* is indeed a suitable host for the herbivore. However, the greenhouse no-choice bioassay revealed a substructure in the pattern and degree of beetle feeding (Table 3). Significantly more ‘Success PM’ plants had no leaf damage (0% leaf defoliation) than ‘Golden Zucchini’ plants (Fisher’s exact two-tailed test, *P*<0.0001). Intriguingly, significantly more ‘Success PM’ plants sustained what we termed ‘extreme’ leaf damage, (⩾80% leaf defoliation) than ‘Golden Zucchini’ plants (Fisher’s exact two-tailed test, *P*=0.004).

## Discussion

We have elucidated a significant factor affecting the structure of striped cucumber beetle (*A. vittatum*) preference within *C. pepo* by quantifying beetle damage on plants of two economically important subspecies, *C. pepo* subsp. *pepo*, and *C. pepo* subsp. *texana*. Our cultivar panels represented the range of the cultivated crop within each subspecies,^34^ and are consistent with reports on the genomic differentiation of these subspecies.^29,30^ Overall, in the variety of bioassays conducted, we found that *C. pepo* subsp. *pepo* cultivars were more heavily damaged by the striped cucumber beetles, and that *C. pepo* subsp. *texana* cultivars were less damaged when presented in choice (Figure 1) or no-choice (Figure 2) experiments. In all, these results strongly implicated subspecies as a decisive driver of *A. vittatum* preference within *C. pepo*.

Previous work examined differential cucumber beetle preference within a variety of cucurbit crops.^1,11,36^ Researchers have employed various levels of structure in which cucurbit plants are tested: some group by species,^11,39–41^ or market class^5,6^ or cultivar^24,36^ within a single species, like *C. pepo*. However, applying this phylogenetic framework to previous studies demonstrates that subspecies is predictive of beetle damage in a variety of experimental designs and cultivar selections. Ferguson *et al.*^11^ conducted replicated field trials of many *Cucurbita* crops, with a heavy representation of eighteen *C. pepo* cultivars. If these cultivars are grouped into subspecies using information from the seed trade and online databases (for example, http://cuke.hort.ncsu.edu/cucurbit/wehner/vegcult/vgclintro.html), then the *C. pepo* subsp. *pepo* cultivars sustained significantly more damage. This is also true of trial results for feeding damage published by McGrath,^6^ where 10 *C. pepo* cultivars were grown among a variety of cultivated cucurbit crops. In addition, when Hoffmann *et al.*^5^ focused on solely *C. pepo* cultivars, throughout the trial, the number of beetles per plant, number of infested plants and defoliation ratings, are again substantially higher in *C. pepo* subsp. *pepo* cultivars. In all of these field trials, authors relied on natural populations of beetles for damage, and reported the presence of multiple beetle species of the tribe Luperini.^6,7,11^ A smaller study,^36^ where four *C. pepo* cultivars—incidentally two from each subspecies—were grown in greenhouse trials with beetles separated by species, also upholds the validity of organizing *A. vittatum* preference by subspecies. Wiseman *et al.*^36^ reported significantly higher stem and cotyledon injury to *C. pepo* subsp. *pepo* cultivars by *A. vittatum* adults alone.^36^

The plant metabolic processes driving beetle preference in *C. pepo*, however, are not well characterized. Broadly within the Cucurbitaceae family, cucurbitacins have been classically described as the key biomolecules dictating herbivore behavior. Generalist herbivores tend to perform poorly on cucurbitacin-rich plants; for instance, two-spotted mites (*Tetranuchys urticae* Koch.) suffered higher mortality,^28^ and had lower fecundity^42^ when feeding on bitter cucumber. In contrast, over the course of a long co-evolutionary history, specialist herbivores of the Cucurbitaceae, like *A. vittatum* and its close relatives in the Luperini tribe (like the spotted cucumber beetle, *Diabrotica undecimpunctata howardi*), have adapted to tolerate cucurbitacins, and in some cases, find them to be feeding stimulants.^17,18,43^ However, there is no strong evidence to support that differences in *A. vittatum* preference between subspecies in *C. pepo* can be attributed to the single qualitative factor of the differential presence of cucurbitacins.

Cucurbitacins B, D and E have been detected in *C. pepo*,^11,19,20,24,44,45^ but they are at extremely low concentrations compared with wild species, and cultivated species known to attract *A. vittatum*, such as several cultivars used for trap cropping.^11,19,46^ Even though cucurbitacins have been detected in *C. pepo*, most of the few precise measurements that exist were taken in root^20^ and cotyledon tissue,^11,24^ not leaf tissue. Cucurbitacins in leaves were found to reach a maximum of 5–10 μg g^−1^ in fresh tissue in a preferred *C. pepo* subsp. *pepo* zucchini cultivar, Black,^44^ which is below the reported level of detection by diabroticite beetle feeding of 20 μg g^−1^ of cucurbitacins in fresh tissue.^19^ Moreover, cucurbitacin content in the leaves of *C. pepo* plants is poorly correlated with cotyledon cucurbitacins, and thought to be controlled by a different genetic pathway.^11^ The genetic basis of cucurbitacin production in *C. pepo*, in general, is also not well characterized. Unlike production of cucurbitacin C in *C. sativus*, cucurbitacin production in *C. pepo* is not controlled by a single gene.^24,47^

In addition, the foundation of most knowledge of the role of cucurbitacins in influencing the behavior of diabroticite beetles is known from Luperini beetle species other than *A. vittatum,*^11,17–19^ most often *D. undecimpunctata howardi.*^11,17–19^ Although these cucurbit specialist beetles are often discussed interchangeably,^5^ there are important differences. For instance, *A. vittatum* tends to excrete less and sequester more cucurbitacins^48^ than its close relatives, and is far less sensitive to cucurbtaicins.^18,19^ Specifically, *A. vittatum* have been shown to react to 0.3 μg pure cucurbitacin B, whereas *D. undecimpunctata howardi* is sensitive at a threshold of over two order of magnitudes lower, 0.001 μg of pure cucurbitacin B.^19^ Finally, behavioral studies also indicate that *A. vittatum* is not reliant on cucurbitacins. The preference of *A. vittatum* for cucurbitacin C present in *C. sativus*, is also inconsistent throughout its lifecycle,^23^ suggesting cucurbitacins may not have a critical role in preference. In addition, these cucurbitacins are neither a prerequisite, nor even augment, *A. vittatum* aggregation pheromone production—an important behavior implicated in increased pest pressure.^21,22^ Overall, from examining both *C. pepo* and *A. vittatum*, cucurbitacins alone do not appear to be a likely candidate for the biochemical basis of preference.

Although cucurbitacins are certainly the most studied specialized defensive metabolite from cucurbit crops, there is evidence that other classes of non-volatile and volatile biomolecules may explain the disparities in herbivore preference between subspecies. Differences in non-volatile nutritive compounds like cotyledon sugar content,^24^ and root nitrogen,^20^ have been reported. Contrasts in both floral^25–27^ and non-floral^3^ plant volatiles have also been shown to elicit differential beetle preference. Other yet unknown factors may exist as well that drive the extreme difference in preference considering that these two *C. pepo* subspecies are genetically distinct,^29,30^ and likely arose from different domestication events.^31–33^ Although future work should explore the broad range of biochemicals including nutrients, cucurbitacin types and overall levels, and volatiles—and the interactions between them—that may drive *A. vittatum* preference, it is important to recognize that preference is likely controlled by a complex array of factors within a dynamic, and environmentally sensitive, biochemical system.

Although efforts will be directed at achieving a more complete understanding of the biochemical and genetic framework that underlie preference, this knowledge of *A. vittatum* preference being structured by subspecies in *C. pepo* will be immediately applicable in plant breeding and management decisions to benefit growers. Trap-cropping strategies already take advantage of highly preferred cucurbits to pull beetles away from the market crop.^49–52^ A derivation of trap-cropping, a push–pull management system,^53^ has been suggested for *A. vittatum* control in *C. pepo*,^27^ where a non-preferred cultivar is paired with a highly preferred trap crop. Our results both identify *C. pepo* subsp. *texana* crops as a the non-preferred ‘push’ planting and prompted the question of whether the ‘push’ factor of the non-preferred cultivar can be strong enough alone to be an effective form of pest management. Specifically, how would *A. vittatum* respond if they were presented with no choice of food source on a farm-scale (for example, ‘Success PM’ monoculture)? In the absence of a ‘preferred’ food source, would the beetles still inflict minimal damage on the ‘non-preferred’ food source?

Our results from both field and greenhouse trials indicate that, although non-preference does not equate to resistance *per se*, it exploits a nuance in striped cucumber beetle feeding patterns, which leads to the non-preferred cultivar sustaining significantly less damage, even when a preferred cultivar is not locally available. In addition, in the greenhouse, concentrated damage on only a few plants of the non-preferred cultivar, were also observed, inviting the question of the role of host plant resistance in non-preference. This result, *A. vittatum* non-preference being manifested both in the presence and absence of a preferred genotype, is likewise corroborated by another no-choice trial of *A. vittatum* in cucumbers (*Cucumis sativus*).^1^

Although these no-choice tests only made use of two of the many available cultivars, by employing the structural framework for organizing *A. vittatum* preference by subspecies within *C. pepo*, countless other cultivars can be rigorously tested in both no-choice and push–pull settings. Overall, the no-choice component of this study has provided new insights into striped cucumber beetle preference and the effectiveness in field deployment, and informs the breeding of *C. pepo* cultivars by providing evidence for the usefulness of introgression of non-preference across subspecies barriers.

This study was the first to consider subspecies as a driver of beetle preference within *C. pepo*, and provided a clear demarcation in preference between the two major commercial subspecies—*C. pepo* subsp. *pepo* and *C. pepo* subsp. *texana*—with *C. pepo* subsp. *texana* being more strongly non-preferred both alone, and among preferred *C. pepo* subsp. *pepo* cultivars. By basing this work in a biological context, this contrast provides an accessible structural framework for understanding variation in preference within *C. pepo*, and applying it in an agricultural setting has been discovered. This structure immediately allows plant breeders and entomologists to augment pest control options, and also promotes future work to understand the biochemical and genetic foundation of *A. vittatum* preference.

## Figures and Tables

**Table 1:** Striped cucumber beetle damage in field and greenhouse (GH) trials of early-harvest *C. pepo* cultivar panel

*Subspecies*	*Damage*^a^^,^^b^				*Market class*	*Damage*^a^^,^^c^				*Cultivar*^d^^,^^e^	*Seed source*^f^	*Damage*^a^^,^^c^			
	*Field*		*GH*			*Field*		*GH*				*Field*		*GH*	
*C. pepo* subsp. *texana*	5.8	a	2.5	a	Crookneck	5.6	a	2.0	a	Dixie^α^	SM	5	ab	2	ab
										Gentry	JS	6	ab	2	a
					Scallop	5.7	a	2.9	a	Golden Bush Scallop^α^	SO	6	a–c	2	ab
										Flying Saucer	JS	6	ab	4	ab
					Straightneck	6.0	a	2.7	a	Cougar	HS	5	a	2	a
										Early Prolific Straightneck^α^	SO	5	a	1	a
										Success PM	CU	5	a	2	ab
										Superpik^β^	TS	5	a	2	ab
										Zephyr	JS	5	a	5	ab
										Slick Pik	JS	7	ab	5	ab
										Lioness	SC	10	a–c	1	a
*C. pepo* subsp. *pepo*	28.2	b	11.3	b	Cocozelle	22.8	b	13.6	b	Cocozelle	HM	22	a–d	12	a–c
										PMR Costata	CU	23	a–e	19	bc
										Costata Romanesco	HM	24	b–e	10	a–c
					Vegetable Marrow	30.1	bc	7.8	ab	Harukan^α^	SC	21	a–d	4	ab
										Magda^α^	CU	40	de	11	a–c
					Zucchini	31.5	c	12.6	b	Partenon	JS	20	a–d	10	ab
										Golden Arrow	FS	21	a–d	9	ab
										Cashflow	SY	25	a–e	13	a–c
										Dunja	JS	27	a–e	14	a–c
										Zucchini Elite^α^	HS	28	a–e	26	c
										Black Beauty^α^	TS	29	b–e	12	a–c
										Reward	OS	29	b–e	13	a–c
										Tigress	JS	31	c–e	8	ab
										Gold Rush	OS	35	de	9	ab
										Goldy	JS	37	de	13	a–c
										Midnight Lightning	HM	39	de	10	a–c
										Golden Zucchini	CU	42	de	12	a–c
										Romulus^αβ^	CU	46	e	14	a–c

Abbreviations: CU, Cornell University produced seed, Ithaca, NY, USA; FS, Fedco Seeds, Waterville, ME, USA; HS, Harris Seeds, Rochester, NY, USA; HM, High Mowing Organic Seeds, Wolcott, VT, USA; JS, Johnny’s Selected Seeds, Winslow, ME, USA; OS, Osborne Seed Company, LLC, Mount Vernon, WA, USA; SC, Seeds of Change, Rancho Dominguez, CA, USA; SM, Seminis, St Louis, MO, USA; SO, Southern Exposure Seed Exchange, Mineral, VA, USA; SY, Syngenta Seeds, Inc., Minnetonka, MN, USA; TS, Territorial Seed Company, Cottage Grove, OR, USA.

a Damage is reported as the least mean square average percent defoliation.

b Means followed by different letters are significantly different at *P*<0.001 using Student’s *t*-test.

c Means followed by different letters are significantly different at *P*<0.05 using a Tukey’s HSD test.

d *n*=15 for field trials unless indicated by ^α^ symbol: ‘Dixie’ *n*=5; ‘Golden Bush Scallop’ *n*=9; ‘Early Prolific Straightneck’ *n*=14; ‘Harukan’ *n*=12; ‘Magda’ *n*=12; ‘Zucchini Elite’ *n*=12; ‘Black Beauty’ *n*=14; ‘Romulus’ *n*=13.

e *n*=12 for greenhouse trials unless indicated by ^β^ symbol: ‘Superpik’ *n*=14; ‘Romulus’ *n*=11.

f Letters represent source of seed.

**Table 2:** Striped cucumber beetle damage in field and greenhouse (GH) trials of mixed-harvest *C. pepo* cultivar panel

*Subspecies*	*Damage*^a^^,^^b^				*Market class*	*Damage*^a^^,^^c^				*Cultivar*^d^^,^^e^	*Seed source*^f^	*Damage*^a^^,^^c^			
	*Field*		*GH*			*Field*		*GH*				*Field*		*GH*	
*C. pepo* subsp. *texana*	3.7	a	3	a	Scallop	3	a	4.8	ab	Golden Bush Scallop^β^	SO	1	a	3	ab
										Flying Saucer	JS	3	a	9	a–c
										Yellow Scallop^β^	SW	3	a	5	a–c
										Woods Prolific Bush Scallop^β^	SE	5	a	2	ab
					Straightneck	3.7	a	0.8	a	Cougar^α^	HS	3	a	1	a
										Multipik^β^	HS	3	ab	1	a
										Superpik	HS	3	a	1	a
										Early Prolific Straightneck^αβ^	SO	4	ab	0	a
										Success PM^α^	HM	5	ab	1	ab
					Acorn/Delicata	4.3	a	3.6	ab	Honey Bear	JS	2	ab	3	ab
										Jester	JS	2	ab	2	ab
										Sugar Loaf^αβ^	NG	3	ab	2	ab
										Royal Ace^αβ^	HS	4	ab	2	ab
										Bush Delicata	CU	4	a	1	ab
										Zeppelin	WG	5	ab	7	a–c
										Honeyboat	OS	6	ab	10	a–c
										Sweet REBA^β^	CU	9	a–c	2	ab
*C. pepo* subsp. *pepo*	23.3	b	10.9	b	Pumpkin	12.3	b	8.2	bc	Racer^β^	JS	9	a–c	9	a–c
										Triple Treat^β^	BR	10	a–c	9	a–c
										Magic Lantern	HS	11	a–c	5	a–c
										Aladdin	HS	14	a–d	9	a–c
										Howden	HS	20	b–e	9	a–c
					Zucchini	34.4	c	13.7	c	Dunja	JS	22	c–e	10	a–c
										Reward^β^	OS	32	de	13	a–c
										Black Beauty^αβ^	TS	32	de	4	a–c
										Zucchini Elite	HS	33	e	20	bc
										Golden Zucchini	SE	54	f	22	c

Abbreviations: BR, Burpee, Warminster, PA, USA; CU, Cornell University produced seed, Ithaca, NY, USA; HS, Harris Seeds, Rochester, NY, USA; HM, High Mowing Organic Seeds, Wolcott, VT, USA; JS, Johnny’s Selected Seeds, Winslow, ME, USA; NG, Nichols Garden Nursery, Albany, OR, USA; OS, Osborne Seed Company, LLC, Mount Vernon, WA, USA; SE, Seed Savers Exchange, Decorah, IA, USA; SO, Southern Exposure Seed Exchange, Mineral, VA, USA; SW, Sow True Seeds, Asheville, NC, USA; TS, Territorial Seed Company, Cottage Grove, OR, USA; WG, Wild Garden Seed, Philomath, OR, USA.

a Damage is reported as the least mean square average percent defoliation.

b Means followed by different letters are significantly different at *P*<0.001 using Student’s *t*-test.

c Means followed by different letters are significantly different at *P*<0.05 using a Tukey’s HSD test.

d *n*=15 for field trials unless indicated by ^α^ symbol: ‘Cougar’ *n*=9; ‘Early Prolific Straightneck’ *n*=14; ‘Success PM’ *n*=14; ‘Sugar Loaf’ *n*=7; ‘Royal Ace’ *n*=14; ‘Black Beauty’ *n*=14.

e *n*=12 for greenhouse trials unless indicated by ^β^ symbol: ‘Golden Bush Scallop’ *n*=11; ‘Yellow Scallop’ *n*=9; ‘Woods Prolific Bush Scallop’ *n*=11; ‘Multipik’ *n*=11; ‘Early Prolific Straightneck’ *n*=10; ‘Sugar Loaf’ *n*=7; ‘Royal Ace’ *n*=11; ‘Sweet REBA’ *n*=10; ‘Racer’ *n*=10; ‘Triple Treat’ *n*=10; ‘Reward’ *n*=6; ‘Black Beauty’ *n*=8.

f Letters represent source of seed.

**Table 3:** Distribution of striped cucumber beetle damage to leaves in greenhouse no-choice bioassay

*Cultivar*	*Subspecies*	*Mean damage*^a^^,^^b^	*Grouped by no damage*^a^^,^^c^^,^^d^		*Grouped by high damage*^a^^,^^c^^,^^e^	
			*0%*	*>0%*	*<80%*	*⩾80%*
Success PM	*C. pepo* subsp. *texana*	29.8% (NS)	24.2%	75.8%	91.3%	8.7%
Golden Zucchini	*C. pepo* subsp. *pepo*	30.6% (NS)	3.9%	96.1%	97.4%	2.6%

Abbreviation: NS, not significant.

a Damage reported as mean percent leaf defoliation.

b No significant difference in mean leaf defoliation between cultivars was detected by a Student’s *t*-test.

c Numbers represent the percent of plants of that genotype with the indicated level of damage.

d No-choice contingency table significant at *P*<0.0001 in two-sided table probability from Fisher’s exact test.

e No-choice contingency table significant at *P*<0.01 in two-sided table probability from Fisher’s exact test.
